# Role of peer-tutors with dementia in Recovery College dementia courses: an ethnographic account

**DOI:** 10.1093/geront/gnag010

**Published:** 2026-02-15

**Authors:** Linda Birt, Melanie Handley, Juniper West, Jarin Alam, Fiona Poland, Esme Moniz-Cook, Emma Wolverson, Geoff Wong, Corinna Hackmann, Bonnie Teague, Rachael Litherland, Christopher Fox, DiSCOVERY Study Team, Ruth Mills, Kathryn Sams, Leanne Hague, Claire Duddy, Charlotte Wheeler, Maria Sanchez, Robert Kelly

**Affiliations:** School of Healthcare, University of Leicester, Leicester, United Kingdom; School of Health Sciences, University of East Anglia, Norwich, United Kingdom; Centre for Research in Public Health & Community Care, University of Hertfordshire, Hatfield, United Kingdom; Research and Development, Norfolk and Suffolk NHS Foundation Trust, Norwich, United Kingdom; Research and Development, Norfolk and Suffolk NHS Foundation Trust, Norwich, United Kingdom; School of Health Sciences, University of East Anglia, Norwich, United Kingdom; Faculty of Health Sciences, University of Hull, Hull, United Kingdom; Geller Institute of Ageing and Memory, University of West London, London, United Kingdom; Research and Publications, Dementia UK, London, United Kingdom; Nuffield Department of Primary Care Health Sciences, University of Oxford, Oxford, United Kingdom; Research and Development, Norfolk and Suffolk NHS Foundation Trust, Norwich, United Kingdom; Research and Development, Norfolk and Suffolk NHS Foundation Trust, Norwich, United Kingdom; Department of Clinical Psychological and Psychological Sciences, University of East Anglia, Norwich, United Kingdom; MSc Innovations in Dementia CIC, Exeter, United Kingdom; (Psychiatry Dementia) Medical School, University of Exeter, Exeter, United Kingdom

**Keywords:** Identity, Peer support, Safe uncertainty, Hope, Realist

## Abstract

**Background and Objectives:**

Receiving a diagnosis of dementia impacts life plans and can lead to feelings of hopelessness and social disengagement. Postdiagnostic support can help people adjust to and assimilate a changing identity. Recovery Colleges in the United Kingdom offer a specific form of postdiagnostic peer-led support. This paper aims to provide a rich account of “stand out” moments where the key tenets of recovery-focused postdiagnostic support were enacted.

**Research Design and Methods:**

Using ethnographic observations and interview data from the *anonymized Study*, a realist evaluation of Recovery College dementia courses, we examined data to specify activities of peer-tutors and the mechanisms that shaped outcomes for people with dementia.

**Results:**

Five Recovery College dementia courses were observed across four NHS mental health services in England. Postcourse interviews were undertaken with 13 tutors (three peer-tutors with dementia) and 32 attendees (eight people with dementia). We found that through co-facilitation of recovery-focused content by peer-tutors who have well developed facilitation skills, attendees appeared to mediate self-stigma, manage emotional uncertainty, and make meaningful social connections in ways which engendered hope for their future.

**Discussion and Implications:**

Identifying the activity between peer-tutors with dementia and course attendees foregrounds key strengths and limitations of this distinctive form of postdiagnostic support. Future work should evaluate longer term outcomes for people with dementia attending recovery courses before potentially expanding this form of postdiagnostic support.

## Background and objectives

Being diagnosed with dementia has a life-changing impact on a person and their family. People may need to adjust to and potentially assimilate a changed identity as a person with dementia (Birt et al., 2017). There is no cure and newly licensed medicines have limited effect (Walsh et al., 2024). Numbers of people with dementia are increasing globally (Wittenberg et al., 2019), and within the United Kingdom, National Health Service (NHS) referral rates to diagnosis in memory services are expanding, meaning there is urgent need to provide sustainable postdiagnostic support. Sustainability in this context is defined as: “… patient-centred, focused on recovery, self-monitoring and independent living, and actively reduces the need for intervention” (Jethwa et al., 2024, p. 5). The period immediately following diagnosis is critical in enabling people to maintain a non-stigmatized social “self” (Birt et al., 2020) and to build personal resilience (Windle et al., 2023) for coping with the combined psychological, physical, social, and emotional effects of this progressive and life-limiting diagnosis (Watts et al., 2014).

Peer-led education offers ways for people to actively adapt and problem-solve to live positively with dementia (Bamford et al., 2021; Jopling, 2017). The U.K. policy identifies key areas for improving the quality of postdiagnostic support, including shifting focus to “living well” with long-term conditions. A national strategy promoted dementia peer-support and learning networks, specifying an objective to empower people with dementia to make their own choices in future planning (Department of Health, 2009). This vision contrasts with lived experience reports of postdiagnostic support disempowering individuals, defined as Prescribed Disengagement (Low et al., 2018). Dementia peer-support initiatives are commonly available world-wide, they are often community-led and, as such, may provide support but will not necessarily specifically enable self-reflection, learning, and empowerment. Finding ways for people with dementia to activate their personal strengths and build positive relationships is especially important as there are variable levels of statutory postdiagnostic support (Dooley et al., 2025).

### Peer support through Recovery Colleges

Recovery Colleges are an international initiative to help people assimilate mental health diagnoses on their own terms and learn ways to live positively with their symptoms (Leamy et al., 2011). The delivery of psychoeducation and adult learning is premised on co-production between people with lived experience and health professionals. Courses focus on personal recovery, informed by the “CHIME” framework tenets: Connection, Hope and optimism, Identity, Meaning and purpose, and Empowerment (Leamy et al., 2011). Where and how Recovery College courses are provided differ, but in the United Kingdom, many align with and complement statutory mental health services (see Supplementary File 1). Importantly, the Recovery College provides flexible access to psychoeducation, peer-support and knowledge sharing from lived experiences in the postdiagnosis period. Courses are generally open access enabling people with lived experience, family or friend supporters and healthcare staff to self-enroll without having to go through a referral system (Perkins et al., 2012). While the term recovery may be at odds with the reality of an illness that reduces life span and quality of life, in this postdiagnosis setting it is used in the context of making sense of what happened (here in the diagnosis) and finding new ways to live a meaningful life with the illness (Perkins et al., 2012).

### Recovery Colleges and dementia

Courses specifically designed by and for people with dementia and their supporters are being added to the Recovery College offer. Nonetheless, such courses are not available in all NHS mental health services (Lowen et al., 2019), and when they are, staff involved in diagnostic services often do not routinely signpost newly-diagnosed people to Recovery Colleges (Wolverson et al., 2024). Where dementia courses are provided the co-production and co-delivery between staff and people with lived experience is considered important to personal recovery from a dementia-diagnosis (Kenny et al., 2016; West et al., 2022). However, there is limited empirical evaluation, including of the causal mechanisms underlying this. A realist review reporting on Recovery College dementia courses led to initial program theories on aspects relating to co-producing courses (Handley et al., 2024). The review results emphasized the need for co-production, tackling stigma, embedding of personal recovery principles, and the importance of providing practical and emotional support to ensure people adjusting to a diagnosis of dementia can access and benefit from the courses (Handley et al., 2024). However, the review found little evidence on the experiences of those who attended these dementia courses.

## Research design and methods

In the DiSCOVERY Study we conducted a realist evaluation of English mental health service delivered Recovery College courses using a case study design (Birt et al., 2023). Realist evaluation is used to identify program theories explaining how interventions are thought to work for various stakeholders by formulating evidence-based program theories. These theories set out how relationships between the underlying mechanisms and contextual factors lead to different outcomes (Pawson & Tilley, 1997).

Important in realist approaches is a detailed understanding of context. Context may be shaped by organization policies and rules, but is also impacted by cultural concepts such as intersubjective beliefs, norms, and values. Collecting data through a focused ethnography, using a case study design, enables examination of cultural factors at play (Jones-Hooker & Tyndall, 2023).

This paper draws on data from ethnographic observations and realist interviews with tutors and attendees who had dementia. However, given that courses took place in group settings, where interactions observed could be supportive or dismissive, we also draw on relevant data from family supporters and staff attendees to specifically examine and define key outcomes for people with dementia in co-producing, facilitating, and attending Recovery College dementia courses.

### Study aim

The realist evaluation aimed to define *what works for whom, in what circumstances, and why*, in Recovery College dementia courses, specifically focusing on outcomes relating to postdiagnostic adjustment. Results on how the evaluation refined initial program theories developed during our realist review is published elsewhere (Birt et al., 2025). This paper has a specific focus on how people with dementia, either in a peer-tutor role or as an attendee, experience Recovery College dementia courses and discusses observed and reported outcomes.

### Ethical approval

Ethical approval was granted by Coventry & Warwickshire Research Ethics Committee (22/WM/0215). This work is part of the DiSCOVERY research program (reference NIHR131676, 2022-2024). Full protocol details are available at Birt et al. (2023). A person with dementia from the DiSCOVERY Partners in Research group attended the ethics committee review meeting alongside researchers.

### Recruitment and sample

Recovery Colleges within English statutory mental health services offering dementia courses were approached. Inclusion criteria were (1) staff, people with dementia, family supporters either co-producing material and/or co facilitating courses, and (2) course attendees—predominantly people with dementia and family supporters but also health and social care staff and members of a Recovery College. Purposive sampling enabled us to recruit courses from diverse geographical locations that had a variety of delivery styles. We sent study information to each site and held discussions before seeking consent from potential participants. In each case, staff and peer-tutors gave written consent to the course being observed. Recovery College staff sent study information to people enrolled on the course at least 48 hr before and course attendees were advised researchers would be present. Attendees’ consent was secured before the course started; all people with dementia were able to give informed consent. Tutors and attendees were informed of the opportunity to take part in a postobservation interview for which we obtained their additional consent. Recovery College staff and dementia service managers were also offered an interview.

### Data collection

#### Focused ethnography

Two of three female researchers (L.B., M.H., J.W.) observed each course; all had extensive experience of research with people with dementia. Observations aligned with ethnographic methods using short, focused field visits (Wall, 2015). At times, researchers joined in activities such as icebreakers, but mostly they sat among the attendees discreetly observing and recording activities. A formal template was not used but notes recorded the setting, actors, materials shared, who led discussions, tutors and attendees’ reactions to materials, chat between actors and changes in attendees demeanor, or involvement. Following each session, researchers debriefed and reflected together.

#### Realist interviews

Tutors and attendees were offered individual interviews following the courses, either virtual or in-person. Topic guides (see Supplementary File 2) were developed with stakeholder advisory groups (Manzano, 2016) drawing on findings from the realist review (Handley et al., 2024). Interviews served the dual purpose of understanding individual experiences of Recovery College dementia courses and testing initial theories of what worked and why.

### Data analysis

Data were pseudonymized. Interviews transcribed verbatim from digital recording and identifiers removed to maintain anonymity. Handwritten field notes were typed up and a single document agreed for analysis; reflective memos were maintained. Data were managed in NVivo (QSR International Ltd., 2020) and analyzed using realist logic (Pawson & Tilley, 1997). Coding was deductive (informed by the review’s initial program theories), inductive (derived from the collected data), and retroductive (inferences about mechanisms based on interpretations of data about underlying causal processes). L.B., M.H., and J.W. then independently revisited coded data on NVivo to develop Context–Mechanism–Outcome–configurations (CMOCs) which set out causal explanations of what works, for whom, when, and how. Twenty CMOCs were grouped to the key concepts they represented (see Supplementary File 3).

An important step in realist evaluation is to consider formal (or substantive) theories that provide underpinning explanations for how mechanisms work in specific contexts to lead to outcomes. The conceptual personal recovery CHIME framework (Leamy et al., 2011) and theories underpinning a person-centered approach to dementia (Downs & Lord, 2017) were identified. Research team meetings identified further conceptual models that explain the value of collaborative and interactive processes in Recovery College dementia courses. These included theories of hope (Dufault & Martocchio, 1985) and how people with dementia create meaning in everyday social interactions.

### Advisory groups

Developing results were structured into prereading discussion papers and slide sets by L.B. and J.W., to enable systematic deliberations with the Partners in Research advisory group (people with dementia) and Staff stakeholder groups, and the wider research team. This iterative process of data analysis continued at regular intervals throughout the 18-month data collection period. Data were enriched with reflections from those stakeholders who gave consent to recording of meetings and use of illustrative quotes. Meeting transcripts were date-stamped, reviewed, and advisory group statements validating or contesting researcher interpretations were highlighted. Specifically, the experiences of the Partners in Research group related to co-producing and attending similar “living with dementia” courses providing triangulation of the empirical data set.

## Results

### Sample

Five recovery-focused dementia courses were observed in four NHS Mental Health Trusts. Four courses were fully embedded within their Recovery Colleges. A fifth course, although originally developed within the Recovery College, was now being delivered as part of NHS postdiagnostic provision. Twenty-three people with dementia were observed and of those, 11 (three peer-tutors [two females]; eight attendees [seven females]) were interviewed across the five case studies (see Table 1 for sample details). To protect site and participant anonymity, we use minimal detail to refer to participants. This level of anonymity is used as few Recovery Colleges are currently running so if personal characteristics were provided as well as method of delivery, the case site might be identified. There was representation of gender and age across the sample, but limited ethnic diversity.

**Table 1:** Sample characteristics.

	*Case study A*	*Case study B*	*Case study C*	*Case study D*	
*Method of delivery*	In person 2.5 hr 3 sessions over 3 weeks	Online 1 hr 3 sessions over 3 weeks	In person 2.5 hr single session	Online 2 hr Single session	In person 2 hr single session
*Delivery team*	2 staff tutors 1 peer-tutor with dementia 2 peer-tutor family supporters	2 staff tutors 1 peer-tutor family supporter	1 staff tutor 1 peer-tutor with dementia 1 peer-tutor family supporter	1 staff tutor 1 peer-tutor with dementia	1 staff tutor 1 peer-tutor with dementia
*Attendees*	*N* = 9 4 spousal dyads (4 people with dementia) 1 service manager	*N* = 11 2 people with dementia 8 family carers 1 staff member	*N* = 13 4 people with dementia 5 family/friend supporters 1 volunteer 3 support staff	*N* = 20 4 people with dementia 7 family supporters 8 staff 1 support staff	*N* = 33 5 people with dementia 9 family supporter 18 staff members 1 support staff
*Interviews*	*N* = 10 2 staff tutors 1 peer-tutor with dementia 2 peer-tutor family supporters 2 attendees with dementia 2 attendee family supporters 1 attendee service manager	*N* = 7 2 staff tutors 1 peer-tutor family supporter 3 attendee family supporters 1 attendee service manager	*N* = 10 1 staff tutor 1 peer-tutor with dementia 1 peer-tutor family supporter 2 attendees with dementia 2 attendee family supporters 1 attendee volunteer 1 support staff 1 service manager	*N* = 5 1 staff tutor 1 peer-tutor with dementia 2 attendee family supporters 1 attendee staff member	*N* = 12 4 attendees with dementia 6 attendee family supporters 2 attendee staff member

**Figure 1:**
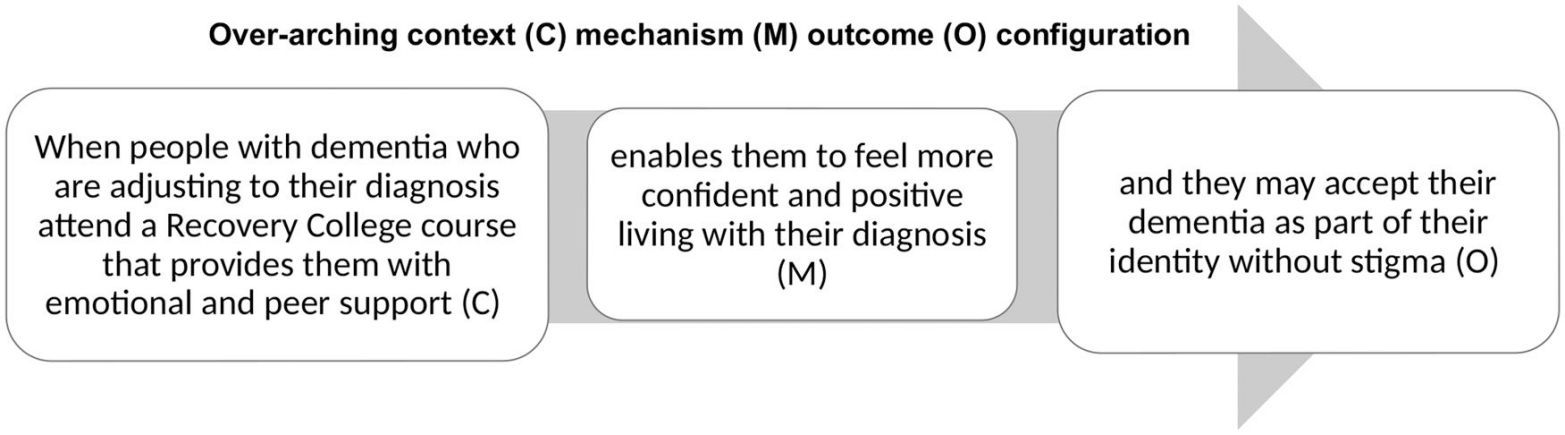
Theme 1: Empowering and enabling non-stigmatized identities.

**Figure 2:**
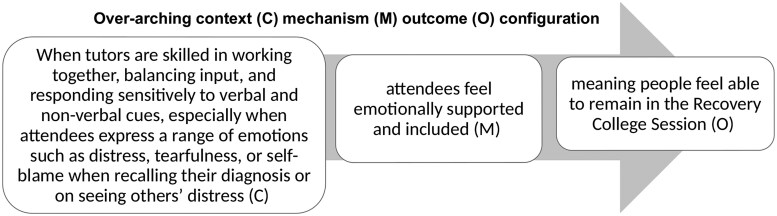
Theme 2: Managing uncertainty for positive reframing.

**Figure 3:**
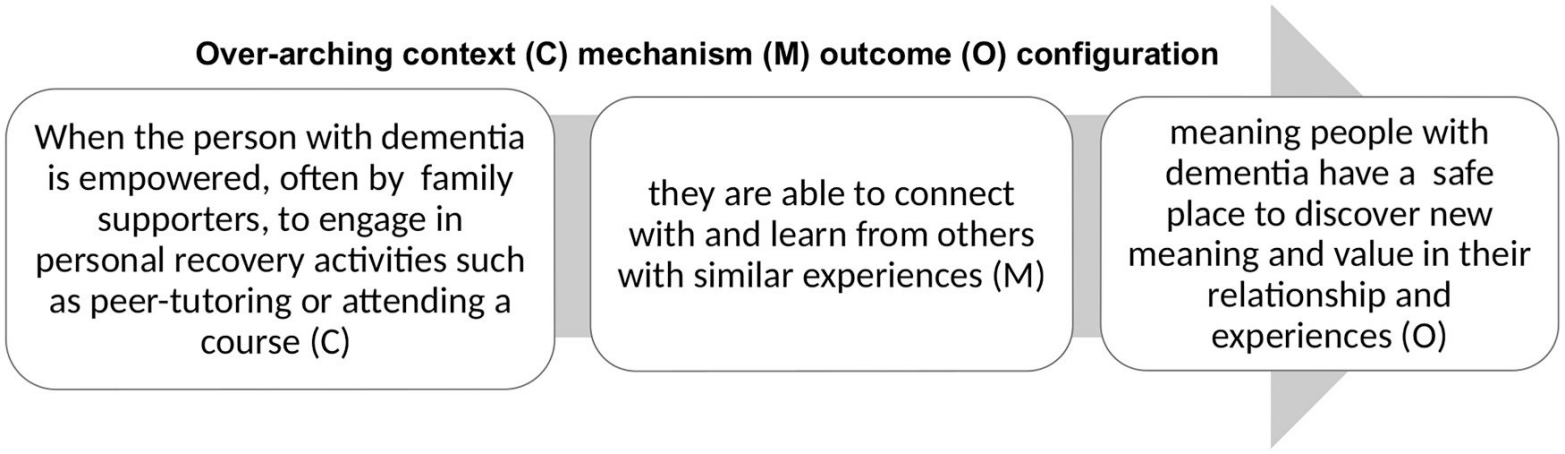
Theme 3: Connecting together enabling hope.

Three thematic areas describe critical “stand out” moments which present how context shapes outcomes for attendees with dementia: (1) empowering and enabling non-stigmatized identities, see Figure 1; (2) managing uncertainty for positive reframing, see Figure 2; and (3) connecting together enabling hope see Figure 3. We present results by providing illustrative examples from observation data, supplemented with interview data, alongside interpretations of the context and mechanisms at play.

### Findings

#### Theme 1: empowering and enabling non-stigmatized identities

An essential component in Recovery College courses is co-production and co-facilitation by peer-tutors with lived experience. The four courses where a peer-tutor with dementia actively co-facilitated, powerfully depicted when the actions of peer-tutors with dementia appeared to enable attendees with dementia to experience positive connections, and importantly to also be seen by others as persons beyond their dementia designation. Essential to inclusivity of course delivery was peer-tutors being “one of the group,” more closely aligned with attendees with dementia than with staff. This happened when the peer-tutor clearly led the course, with the staff tutor merely prompting or helping to keep to time. In all in-person courses, the peer-tutor at some stage positioned themselves physically with the attendees, and in one course the peer-tutor with dementia sat in the circle amongst attendees.

A “stand out” moment occurred during an in-person, 2-hr single session course (Case D). The course was facilitated by a staff tutor and a peer-tutor with dementia; the peer-tutor predominantly led all activities with small prompts from the staff tutor. In this session, all information was shared verbally, unlike other sessions where there were powerpoint slides, handouts, and sometimes writing activities. Attendees included staff, family supporters, and several dyads (person with dementia and family supporter). The peer-tutor, who had early onset dementia, recounted their traumatic experiences of diagnosis, and ways in which they now live their life more positively. This included how they maintain social connections, get involved in research, and find purpose and meaning in everyday activities such as going for walks. Toward the session end, the staff tutor asks attendees to share experiences and for questions. Both tutors are sitting at the front of the room and chairs are set out in five rows with a central gap to walk down. Our observation fieldnotes capture ways in which the peer-tutor empowers an attendee with dementia to have a voice within a large and unfamiliar group of people.

#### Observation Extract 1

A person with dementia sitting in the back row next to his spouse starts to speak, he lifts a hand up, hesitantly stumbling to get words out… “happy with you speech, can’t speak and I want to speak” relating to what the peer-tutor with dementia is saying. The peer-tutor speaks directly to him and validates his experience; “It’s frustrating, it is never easy.” The attendee’s speech grows stronger as the peer-tutor nods towards him, and the attendee says, “that’s exactly what I’m feeling, yes.” The peer-tutor stands and moves forward into the gap between the rows of chairs to speak directly to the attendee, who then talks a bit about his previous occupation, “used to work on a drawing board.” His family supporter joins in to say the attendee might be embarrassed about his disabilities caused by dementia. The peer-tutor moves further forward and their one-to-one exchange continues, encouraged and reassured by the peer-tutor who says “you put together the most wonderful sentence, don’t stop,” “thank you for sharing” and “this is a very touching moment.” Despite the direct interaction, the peer-tutor is simultaneously inclusive of everyone in the room by looking around and using physical presence to draw everyone in to the exchange, getting nods and murmurs of agreement from others. They conclude that people with dementia need more time to process and speak in each moment.

This extract demonstrates how important specific actions can be in promoting a nonstigmatized identity and/or felt sense of self for people with dementia. The actions that created the context (outlined in the overarching CMOC above) were the peer-tutor’s observation of the room that led them to notice a peer struggling to speak and raising their hand; the peer-tutor used active positioning to bring others into what was, in effect, a one-to-one interaction. The outcome was the attendee with dementia positioned himself as a person by occupation, here describing his employment. A wider outcome was learning for others in the room about the importance of giving people with dementia time and attention to speak. On interviewing the peer-tutor with dementia afterward, they reflected on this interaction:

… but he put together this amazing sentence that just shows how, if people are in that environment where they feel safe, they can do more than they think they can. It just relaxes them into, for him, speech. … I mean, we have [standout moments] every time with peer support, but to have it happen with a stranger, it’s always just a magic moment.

These may be examples of transformative learning (Oh, 2013).

By providing examples of living positively, peer-tutors with dementia appeared to help attendees to have hope and consider themselves in more valued ways, thereby potentially reducing self-stigma. The input from peer-tutors across all courses appeared to help family supporters and other attendees to consider people with dementia in nonstigmatizing ways. However, there were occasions where peer-tutors who were family supporters, talked of their experiences in ways that position the care of a person with dementia as burdensome.

In telling their experiences peer-tutors relived stigmatizing experiences and it was important they were empowered to have control over what they shared and when. Here in a postsession interview from Case A, the family supporter provides context to an event where the peer-tutor tells of sharing her diagnosis with a long-standing social group.

#### Observation and Interview Extract 2

***Peer-tutor daughter*** *:* Yeah, you do sometimes in [*share the experience of telling friends*] some of the sessions, but not every session. So, I would say that when mum feels OK to share that she does and other times she doesn’t. (observation noted daughter looked to mother and checked she alright to tell story)

***Peer-tutor female with dementia***: It’s a very difficult thing. They’d all just come back from a group outing and I’d got to tell them because I couldn’t just stand there because they were saying, “How are you?” I had to say, “I’ve got dementia.” There was everyone looking at everybody else and so in the end I just said, “Well we’ll go now then.”

***Interviewer to peer-tutor***: Yeah, so it sounds like each session, there is no script, you don’t always say the same thing, it’s whatever you feel is the right thing at that time.

***Peer-tutor with dementia***: Yes.

Here, we see the family supporter’s work in creating safe spaces for people with dementia either as peer-tutors or attendees to revisit and share previous stigmatizing events. When peer-tutors shared their diagnosis experience, attendees nodded as if agreeing and recognizing their experience was not unique nor stigmatizing.

#### Theme 2: managing uncertainty for positive reframing

People usually attend Recovery College courses within a few months of diagnosis, meaning emotion and shock from receiving their diagnosis can still be raw. This can lead to people becoming distressed during the course. While staff tutors will usually be able to draw on clinical training to support an attendee who is emotionally upset, peer-tutors often do not have formal training for this. This “stand-out” moment illustrates both the empathy peer-tutors can display with others’ lived experiences, and the skills they can adopt to contain upsetting emotions in others.

The course was in-person and a single 2.5-hr session (Case C). The setting was a meeting room in a building used by the mental health service. The course was facilitated by a staff tutor and two peer-tutors: a person with dementia and a family supporter There were 10 attendees: people with dementia attending alone, family supporters attending alone, two dyads (person with dementia and family or friend supporter), and a staff attendee. Attendees heard about the course mainly from their clinician who was the staff tutor, but a couple had heard from other community groups including the attendee who was distressed. Before the course started a person with dementia, attending alone, was visibly distressed, attendees waited with some hesitant anticipation for the course to start, a few people talking to each other. The extract below describes the situation approximately 10 min into the course; the group had introduced themselves by stating they had dementia or were a family or friend supporter.

#### Observation Extract 3

Everyone is sitting around a large central table which means people can have eye contact. The attendee shows small signs of anxiety through worried facial expression. She then becomes distressed and tearful, self-blaming for having dementia. Everyone stops and listens and the peer-tutor with dementia responds and offers reassurances. Following the exchange, the staff tutor takes a seat near to the computer for presenting slides and indicates non-verbally to the support staff member to move to the empty chair next to the person. A group activity is facilitated where peer-tutors hand round a wellbeing questionnaire for attendees to chat through together and complete. There are high levels of engagement by all with ‘ad hoc’ questions being asked about dementia, and some laughing and joking. The attendee again becomes tearful while talking of a traumatic experience, the peer-tutor with dementia takes over from the staff member sitting next to the person. They speak quietly and supportively and the attendee visibly calms. Following slides about the ageing brain, the staff tutor sets the scene for a further interactive activity, by asking, “What is your understanding of what it is like to have dementia? What images come to mind?” The peer-tutor with dementia gets up; *“*Let’s write some of these down” and moves to a flipchart. The attendee again becomes distressed: “It’s my fault I’ve got it [dementia]. I blame myself.” The staff tutor asks the person and the group to focus on dementia symptoms “what sorts of symptoms do you have?” Attendees including the distressed attendee offer words such as “forgetfulness” and the peer-tutor family support writes them on the flipchart, the peer-tutor with dementia talks about his own experience to keep the group joining in.

This example provides evidence of both the staff and peer-tutor being aware of others’ emotions and having skills and being prepared to work together to manage emotions. For example, here the staff tutor indicates for someone to sit next to the attendee and shifts the focus onto symptoms rather than causes. The peer-tutor with dementia showed awareness of the attendees’ struggle and had the confidence to support with quiet interaction. The attendee is able to contain their distress, and the rest of the group can relax. The mechanism found to help resolve this situation appears to be the ability of tutors to create a space of psychological security, where a range of emotions can be acknowledged rather than dismissed. In the postcourse interview, we spoke to the peer-tutor with dementia about the attendee’s distress and how the situation was managed. The peer-tutor explained they helped the attendee contain their emotion: “I was worried. When the lady who presented with the very emotional, tearful response at the beginning, you know, she was on the verge of it being appropriate to remove her from there to somewhere quiet for a one-to-one chat.” They go on to explain that if the staff tutor left the room this would leave the peer-tutors uncertain about what to do: “I don’t know how I would’ve coped with that because the only person at the moment who is competent to deliver the rest of that presentation is the practitioner [staff tutor].” This highlights a challenge for delivering courses if only one or two people have the knowledge or skills to facilitate. Finally, the peer-tutor confides that the situation felt like a “small pressure cooker room” where “hysteria could have taken over in the room.” Importantly, because all tutors were able to effectively help the attendee “hold” their emotion, the person was calm enough to attend to the information being shared by the staff tutor on the potential causes of dementia, and later she is heard to say, “I don’t need to blame myself.”

Further into the session the peer-tutor with dementia leads a section creating opportunities for the attendee with dementia to share positive aspects of herself beyond dementia.

#### Observation Extract 4

The staff tutor asks the peer-tutor with dementia to discuss their experiences, he engages the group, and they are all listening. When speaking of their fundraising, the previously distressed attendee with dementia chips in that she joined the fundraising walk. The peer-tutor passes pictures round of things they have done; there is some laughter as they talk. The peer-tutor describes being able to use “dementia goggles”; “You are valuable as consultants.” The attendee with dementia says “you feel wanted”; there is a visible change in her facial expression and body language appears more upright and smiling, “you meet people who make you laugh.” This stand-out moment demonstrates the value of having peer-tutors with lived experience of dementia who have empathy with emotional responses, but also demonstrate ways in which they have managed the uncertainty of dementia themselves.

#### Theme 3 connecting together enabling hope

While a person with dementia may attend a Recovery College course alone, we observed more people attending as a dyad with a spouse, adult child, or friend supporter. During in-person courses, peer-tutors created content and time for dyads to undertake activities together, prompting connections in a neutral and potentially “safe place” away from usual home life. In Case A, all attended as a partner/spousal dyad; each dyad discussed between themselves what made them feel stressed. We noted phrases such as “when you rush me to go out I feel stressed and anxious.” We observed when activities asked dyads to work together, staff and peer-tutors often moved to be physically present with the dyad and to support conversations between individuals. Sometimes, the format of the Recovery College course led to new understandings between relatives. In this stand-out moment during an in person single 2.5-hr course (Case C), the relationship history between the person with dementia and the adult-child was difficult; a daughter’s family supporter considered their mother to be in denial of their diagnosis, as they had never spoken about having dementia.

#### Observation and Interview Extract 5

Once everyone had settled, the staff tutor stood at the front of the room, welcomed everyone to the session and invited round table introductions. Everyone used the word “dementia” during their introduction although there was no stated prerequisite for this. Each person said why they were there, and the mother of one child/parent dyad clearly said three words “*I have dementia*,” at which the daughter showed complete surprise.

The daughter later explained during an interview how this incident enabled her to understand more of her parent’s experience and to begin to know how her mother felt about their diagnosis:

It was the first time my mum had actually said that she had dementia there. … She’d not said it before, no. … That was the first time and she actually said those words and I was like, “Oh God, I can’t believe it.” … I was really surprised and it made me feel quite emotional. You know, I could have easily burst into tears. … and it probably made me feel more connected to her because it’s difficult to be close to my mum.

The context of a group expectation that the person will identify themselves can be viewed in two ways. It may be interpreted as a nonjudgmental space within the Recovery College course that enabled a person with dementia to verbally acknowledge their dementia-diagnosis in front of their relatives. However, it could be seen as removing the right of the person with dementia to avoid discussing their diagnosis with family. While the daughter found it cathartic, the follow-up interview with the mother was very brief and it appears the decision to attend was not fully her own: “I don’t know, my daughter said she’d take me. I would meet other people.” We do not have data on how comfortable she was with saying she had dementia.

In some courses, we observed delivery of material being tutor-led with limited space or activities for attendees to connect. Factors like the setting and method of course delivery affected whether people had the time and physical space to connect formally or informally. For example, being in a warm and welcoming setting with space for coffee breaks and movement meant people interacted more. Here, a peer-tutor with dementia who delivered courses both online and in person (Case D) explains the importance of such breaks for socially connecting and information sharing.

#### Interview Extract 6

Peer-tutor with dementia: *Also it may be a bit of a bizarre thing but you’d miss the coffee breaks [online] and we have really good conversations in coffee breaks. We will find out, we’ll talk to people and people are open and much more likely to say something that’s concerning them or something they might think it’s silly and don’t want to say in front of anyone else. All sorts of things have come out of previous groups in the coffee break haven’t they. We’ll talk about all sorts with people and you’d miss that.*

In courses run in-person across several sessions, we observed interactions between attendees and peer-tutors increasing over time. Coffee breaks and food sharing led to an attendee with dementia striking up conversation and sharing recipes with another attendee. The person with dementia was thus enabled to present themself as a cake-baker and to discover shared interests with an attendee who was a stranger, socially connecting and empowering conversations beyond having a dementia-diagnosis. Nonetheless, there was an absence of culturally diverse social activities presented or discussed. A family supporter spoke of her exasperation at finding appropriate community activities for her mother, born in the Caribbean, who came to the United Kingdom as an adult. “We searched for things that she could do, but we live a predominantly white area. I contacted the services just to find out what if there is anything that’s specifically for Afro-Caribbean people. I think there’s … but it’s in the daytime and I don’t even know if it’s still running. I don’t think the [recovery] course is set up to even address it.”

We saw activities creating safe spaces for people with dementia to join in without fear of failure or need to share or explain their dementia. In warm up activities prefaced with *tell us why you are here?* people usually stated their dementia status or supporter, or staff role. This created a status divide. In one course, the peer-tutor suggested a more inclusive warm-up: people were asked if they were a dog or cat person. The activity was introduced using slides with images of cats and dogs and the peer-tutor stated their preference. Every attendee was able to contribute, there was laughter, talk of pets from childhood, and when their families had been at home; this prompted shared accounts between spousal and child/parent dyads. If people liked neither cats nor dogs the staff tutor asked about any animal they did like. This led to an attendee with dementia hesitantly telling the group about the birds which they chased with sticks in the garden. They had difficulty with word-finding, but were given time to tell their account. As the group discussed this image and shared laughter, we observed the person sitting a little taller in their chair and looking around the room more actively. The staff tutor started each week reminding people of the previous week’s animal discussion, scaffolding more talk around this nonthreatening topic. These examples demonstrate dementia course activities creating spaces for people to feel included and interconnected to others.

## Discussion and implications

### Summary of findings and comparison with existing literature

The concept of recovery-focused care being appropriate for people with dementia was raised as early as 2010 by Hill. However, ours seems to be the first empirical evaluation to identify the specific contextual and constituent features of Recovery College dementia courses and what outcomes can be for people with dementia in either peer-tutor or attendee roles. Drawing on data collected during a realist evaluation, we consider underlying causal processes, or mechanisms, triggered by varying contextual features of five recovery-focused dementia courses. We do not discuss what works in a mechanistic or evaluative sense as our findings indicate that what works depends on who the person is and context. The mechanisms evident within our CMOCs (Supplementary File 3) and these “stand-out” exemplars can be seen to illustrate theories that align with the CHIME framework, namely, Connectedness, Hope through positive reframing, nonstigmatized Identity and Empowerment. We now discuss these in relation to dementia literature and postdiagnostic support principles.

**Connectedness:** Attending a Recovery College course can enable people with dementia to maintain a social self. By connecting and taking time to inter-relate with others with similar experiences, loneliness in people with dementia can be counteracted. Structuring the course with time for informal talk between attendees and tutors connects people through shared interests or humor, not just through the dementia label. The importance of enabling different relationships beyond “person with lived experience” and “family supporter” has been highlighted in general Recovery College course evaluations (Reid et al., 2020; Toney et al., 2018) and in dementia literature (Birt et al., 2020). Additionally, as co-produced psychoeducation is a collaborative and interactive process that creates value through meaningful exchanges, attendees with dementia can connect despite differing levels of engagement, by interacting verbally or nonverbally, watching and listening. Providing a variety of materials and media with which attendees can connect with may be important for the inclusion of people who have limited literacy or verbal skills. Tutors also need to attend and respond to nonverbal and tentative verbal approaches in order to connect with the person with dementia and bring them into the conversation (Braithwaite Stuart et al., 2022).

**Hope through positive reframing:** Meeting other people with dementia can help individuals reframe their experiences and foster hope. Generating and maintaining hope is an interpersonal process (Dufault & Martocchio, 1985; Miller, 1985) and after a dementia-diagnosis, people may face challenges in adjusting expectations (Wolverson et al., 2010). Comparing their situation with others and learning coping strategies from peers can foster hope (Amponsem et al., 2023). Social interactions create opportunities for experiencing fun and to affirm people’s continuing capacity for positive experiences. Such reciprocity and reaffirming support help generate more hopeful experiences (Jackman et al., 2024). However, dementia is a syndrome of signs and symptoms with lived experiences shaped by individual psychosocial influences including co-morbidities, personal and social resilience, alongside culture and beliefs. In each course in our study, tutors acknowledged complex debilitating symptoms, and carer strain. To uncritically imply that a recovery-focused approach to postdiagnostic support enables people to “live well” across the whole trajectory of the illness risks creating personal and societal narratives that one has failed if contextual factors such as cognitive ability, co-morbidity, relationships, and socioeconomic circumstances restrict a person’s ability to be connected, to have choice, and control. Rather than theorizing recovery in dementia as another feature of successful aging, it is more appropriate to position recovery as an *ongoing process of accommodation and moving beyond what has happened* (Perkins et al., 2016, p. 5). To suggest that a person with dementia can recover a life only if they stay socially connected, continually live meaningful lives, and have control over their life is reductionist. This narrative may lead to a person or family supporter masking physical and cognitive decline and any increasing distress. Such masking, or lack of attention to decline, risks reducing societal empathy and limited access to support.

**Non-stigmatized identity:** Our findings highlight the importance of creating and exemplifying nonjudgmental spaces, nonstigmatizing spaces, where the contributions of people with dementia are of equal value to those of health care staff and family supporters. By sharing and building peer experiences, people with dementia are encouraged to recover a nonstigmatized life after diagnosis (Mukadam & Livingston, 2012). Peer-tutors with dementia bring authenticity to their interactions with attendees, where they can connect through sharing experiences in an immediate, validating, and empathetic way. However, attendees’ experiences will be diverse and intragroup interactions need exploring further. In our study, people were predominantly sign posted to in-person courses but in online courses tutors had to react to unknown attendees. Peer-tutors with dementia were supported to accomplish complex occupational and social activities and they could see the benefits attendees gained, thus potentially increasing peer-tutor’s sense of self-worth and confidence. These findings align with other evidence that people with dementia can and do maintain social connections and are involved in helping others, so sustain a nonstigmatized identity (Birt et al., 2020), affirming self-hood (Hennelly et al., 2021). A Recovery College dementia course is not only a social setting but also one which offers learning about dementia and experiences of how people can navigate life positively with dementia, so replacing guilt and shame with validation and empowerment. However, Reid and colleagues’ (2020) evaluation of a recovery education center noted that deconstructing self-stigma was an ongoing practice. This highlighted for us that while peer-tutors in our study did demonstrate and articulate self-esteem and purposeful activity, this could not reliably evidence longer-term positive outcomes for attendees. Our CMOC (Theme 1) indicated an outcome for people with dementia attending Recovery Colleges was to develop or maintain a stigma-free identity. Further evidence is needed on the longer-term impact of single courses and if longer-term sustainable postdiagnostics recovery-focused support is needed (Moniz-Cook & Mountain, 2023).

**Empowerment through safe uncertainty:** People often attend Recovery College dementia courses quite soon after diagnosis. This can be a time when people feel emotionally unsafe as they face a health threat, and individuals and family supporters experience concerns about each other’s well-being (Moniz-Cook et al., 2006). The learning embedded in Recovery College courses may be essential to help people with dementia gain a sense of emotional safety (Kuske et al., 2021). Recovery-focused and person-centered postdiagnostic support gives control to the person with dementia. The ethos of encouraging people to be empowered and have choice even if these are deemed risky by others (Clarke et al., 2011), may be seen to reflect the theory of “safe uncertainty” articulated by Mason (2022). When under stress, people often wish for certainty and that clear decisions are made for them, but that this in itself may bring negative outcomes (Mason, 2022). A more therapeutic framing of options is to accept there are no definitive solutions or ways to sort the “problem” (here a dementia-diagnosis). Instead, there needs to be a shared process of learning to manage the uncertainty that life brings and to use personal resources to deal with everyday life (Mason, 2022). Such active learning aligns with framing recovery as an ongoing and personal process (Leamy et al., 2011) as Recovery Colleges may empower people with skills to consider ways to manage future challenges (Hill et al., 2010). Having choice, control, and self-determination are all associated with empowerment in people with dementia (van Corven et al., 2021). Mental health experience–based co-production initiatives are designed and shown to support people to make sense of real-life problems and find ways to move forward by learning from the past to shape the future, even when they are uncertain (Palmer et al., 2019).

## Study limitations

We acknowledge the limitation of recruiting from just four sites where our case study ethnographic approach allowed in-depth observations of diverse course delivery styles. Recovery College dementia courses are relatively new and require future research to evaluate the longer-term impact of this form of postdiagnostic support, and the impacts for different people. Specifically, there is more work needed to understand whether the use of the term Recovery is appropriate in the context of postdiagnostic support. We sought, but did not achieve, an ethnically diverse sample despite recruiting from Recovery Colleges in areas with ethnically diverse communities. Known barriers to attending mental health services for both diagnosis and support might explain limited recruitment of non-White British people in this small sample. Another limitation could be the absence of detailed demographics of the sample. We deliberately chose not to record demographic information on those in the observations as we considered this could further highlight the presence of researchers. However, collecting demographic data on those interviewed would have provided greater granular data on the micro-contextual factors of participants. There is a need to consider cultural tailoring for international use with studies undertaken with other groups.

## Conclusion and recommendations

This study is the first to examine co-production, co-facilitated peer support within the context of Recovery College dementia courses—a distinct and novel form of recovery-focused postdiagnostic dementia support. In-depth case study methodology has offered themes associated with positive constructs such as connectedness, hope, empowerment and counteracting a stigmatized identity associated with a dementia-diagnosis. This study reflects a next step in identifying how recovery-focused postdiagnostic support might enable people to have hope for the future following a dementia-diagnosis. Nonetheless, there is scope for more detailed exploration of recovery-focused, postdiagnostic dementia support that should consider the ways in which intragroup interaction occurs and the role of peer support between attendees as well as between peer-tutor and attendees. Trial research could provide an evidence base on efficiency of this form of postdiagnostic dementia support providing empirical evidence as to whether wider implementation is justified. To understand cultural mechanisms, there is scope to compare the United Kingdom with international Recovery College models. We suggest the context mechanism and outcomes outlined in this paper set parameters for future research to design conceptually driven postdiagnostic psychosocial intervention studies in dementia.

## Supplementary Material

gnag010_Supplementary_Data

## Data Availability

The data that support the findings of this study are available on request from the corresponding author (L.B.). The data are not publicly available due to pseudonymized data sets and sharing them may compromise the privacy of research participants.
