# Supplementary material for: Role of peer-tutors with dementia in Recovery College dementia courses: an ethnographic account
Source: Gerontologist. 2026 Feb 15;66(5):gnag010. doi: 10.1093/geront/gnag010 (PMC13082387; doi:10.1093/geront/gnag010)
Supplement: gnag010_Supplementary_Data [file gnag010_supplementary_data.pdf]

Supplementary files: Role of peer-tutors with dementia in Recovery College dementia courses: an ethnographic account

Linda Birt & Juni West

Additional file 1 – Background to Recovery Colleges

Additional file 2 – Topic Guides

Additional File 3: Supporting data on context (C) mechanism (M) and outcome (O) configurations (CMOCs) which support Theme development

Additional File 1: Background to Recovery Colleges
Authors Birt, L., & West, J. (2025)

The first UK Recovery College was set up in 2009, growing to 85 by 2017 (Perkins, Meddings, Williams & Repper, 2018). The model has been replicated internationally with Recovery Colleges now in existence or developing in 22 countries (King & Meddings, 2019). The recovery model is flourishing in adult statutory mental health services, encouraged since 2011 by the Department of Health commissioned 'Implementing Recovery through Organisational Change' (ImROC) collaborative https://imroc.org/. A recovery-focused, peer-led psychoeducation approach is adopted and embedded within strategic care delivery in mental health Trusts, to complement clinical care and improve outcomes beyond a narrow focus on symptom reduction, to help people rebuild meaningful, satisfying lives, despite limitations caused by mental health difficulties (Perkins et al., 2018).

Recovery Colleges operate using five key linked conceptual processes - the CHIME recovery framework – robustly developed to underpin the term recovery in this context: **Connecting** with others, inspiring **Hope**, maintaining a positive **Identity**, finding **Meaning** and purpose in life outside of symptoms and **Empowering** control over life and a focus on strengths (Leamy, Bird, Boutillier, Williams & Slade, 2011). Recovery College courses offer access to distinctive peer support both from co-producing courses and/or attending them (Sommer, Gill & Stein-Parbury, 2018). Recovery Colleges create adult learning environments which moderate power dynamics between service users and staff, to reduce stigma and increase attendees' sense of hope and empowerment (Meddings, Byrne, Barnicoat, Campbell & Locks, 2014; Sommer et al., 2018; Zabel, Donegan, Lawrence & French, 2016). Attendees report developing novel coping strategies, improving self-worth, wellbeing and quality of life (Meddings et al., 2014; Rinaldi, Marland & Wybourn, 2012; Wilson, King & Russell, 2019; Zucchelli & Skinner, 2013). Implementing a recovery-focused approach through Recovery Colleges has been widely evidenced as bringing benefits as well as challenges to access (Allard et al., 2024; Bowness et al., 2023; Whitley et al., 2019).

A typical adult mental health Recovery College offers courses on mental health and recovery, designed to increase attendees' knowledge, skills and confidence in self-management of their own mental health and wellbeing. Courses range from one-off sessions to several sessions spread over a set number of weeks. All courses are co-produced and co-delivered by peer tutors - that is, people with lived/expert experience - and mental health staff. Peer tutors prepare for their role through having training to teach and support, and receive supervision to ensure any sensitive issues can be supported effectively. A theory of change model for Recovery Colleges has been co-developed within adult mental health contexts that identifies four mechanisms of change (Toney et al., 2018) empowering environment - opportunities for choices; shifting balance of power; enabling different relationships and connecting with peers; and facilitating personal growth through shared learning and strength-building. Outcomes include changes in the attendee including improved wellbeing, reinforced by life changes they could observe. This model is highly applicable to enabling desired outcomes for post-diagnostic support in dementia.

The CHIME Recovery framework (Leamy et al., 2011) as operationalised through Recovery Colleges, has clear links with the NICE-recommended person-centred care

framework for dementia (Brooker & Latham, 2016), the Royal College of Psychiatrists Memory Services National Accreditation Programme (MSNAP; Jethwa, Fern, Abhayaratne & Wariabharaj, 2022) and the National Dementia Strategy objective to develop peer support and learning networks (Department of Health, 2009). Key domains for person-centred care are **valuing** people living with dementia and those (both informal family and friends and health and social care staff) who care for them; providing care that is **individualised**; understanding and acting from the **perspectives** of people living with dementia - which can reinforce connections, meanings and identities; and creating positive **social-psychological** environments - which can build hope and empowerment (Brooker & Latham, 2016).

Five recovery processes giving the acronym CHIME

Connectedness with others;
inspiring **Hope** and optimism about the future;
maintaining a positive **Identity**;
finding **Meaning** in life outside of symptoms;
and **Empowerment** with control over life and a focus on strengths. (Leamy et al., 2011)

References

Allard, J., Pollard, A., Laugharne, R., et al. (2024). Evaluating the impact of a UK Recovery College on mental well-being: pre- and post-intervention study. BJPsych open, 10(3), e87. https://doi.org/10.1192/bjo.2023.646

Bowness, B., Hayes, D., Stepanian, K., et al. (2023). Who uses Recovery Colleges? Casemix analysis of sociodemographic and clinical characteristics and representativeness of recovery college students. Psychiatric rehabilitation journal, 46(3), 211–215. https://doi.org/10.1037/prj0000532

Brooker, D., & Latham, I. (2016). *Person-centred dementia care making services better with the VIPS framework (2nd ed.)*. London Philadelphia: Jessica Kingsley Publishers.

Department of health. (2009). *National Dementia Strategy Living well with dementia: A National Dementia Strategy Putting People First.* https://assets.publishing.service.gov.uk/media/5a7a15a7ed915d6eaf153a36/dh_094051.pdf

Jethwa, J., Fern, M., Abhayaratne, C., & Wariabharaj, K. (2022). *Quality Standards for Memory Services Eighth Edition*. https://www.rcpsych.ac.uk/docs/default-source/improving-are/ccqi/quality-networks/memory-clinics-msnap/msnap-standards---8th-edition-(2022).pdf?sfvrsn=d8341549_2

King, T., & Meddings, S. (2019). Survey identifying commonality across international Recovery Colleges. *Mental Health and Social Inclusion,* https://doi.org/10.1108/mhsi-02-2019-0008

Leamy, M., Bird, V., Boutillier, C. L., Williams, J., & Slade, M. (2011). Conceptual framework for personal recovery in mental health: systematic review and narrative synthesis. *British Journal of Psychiatry*, 199(06), 445–452. https://doi.org/10.1192/bjp.bp.110.083733

Meddings, S., Byrne, D., Barnicoat, S., Campbell, E., & Locks, L. (2014). Co-delivered and co-produced: creating a recovery college in partnership. *The Journal of Mental Health Training, Education and Practice*, 9(1), 16–25. https://doi.org/10.1108/jmhtep-04-2013-0011

Perkins, R., Repper, J., Rinaldi, M. and Brown, H.(2012), Recovery Colleges, Implementing Recovery Through Organisational Change, London.

Perkins, R., Hill, L., Daley, S., Chappell, M., & Rennison, J. (2016). 12.'Continuing to be me'– Recovering a life with a Diagnosis of Dementia. Available from https://www.slamrecoverycollege.co.uk/uploads/2/6/5/2/26525995/continuing-to-be-me-recovery-and-dementia-briefing-sept-2016.pdf. Accessed on 1.08.2025

Perkins, R., Meddings, S., Williams, S., & Repper, J. (2018). *Recovery Colleges 10 Years On. ImROC.* https://static1.squarespace.com/static/65e873c27971d37984653be0/t/668c0c17506df25c98d01377/1720454168113/ImROC-Recovery-Colleges-10-Years-On.pdf

Rinaldi, M., Marland, M., & Wybourn, S. (2012). *Annual Report 2011 – 2012 South West London Recovery College*. http://rfact.org.au/wp-content/uploads/2015/05/SW-London-Recovery-College-evaluation-2011_12-v1-0.pdf

Sommer, J., Gill, K., & Stein-Parbury, J. (2018). Walking side-by-side: Recovery Colleges revolutionising mental health care. *Mental Health and Social Inclusion*, 22(1), 18–26. https://doi.org/10.1108/mhsi-11-2017-0050

Toney, R., Elton, D., Munday, E., Hamill, K., Crowther, A., Meddings, S., Taylor, A., Henderson, C., Jennings, H., Waring, J., Pollock, K., Bates, P., & Slade, M. (2018). Mechanisms of Action and Outcomes for Students in Recovery Colleges. Psychiatric Services, 69(12), 1222–1229. https://doi.org/10.1176/appi.ps.201800283

Wilson, C., King, M., & Russell, J. (2019). A mixed-methods evaluation of a Recovery College in South East Essex for people with mental health difficulties. Health & social care in the community, 27(5), 1353–1362. https://doi.org/10.1111/hsc.12774

Whitley, R., Shepherd, G., & Slade, M. (2019). Recovery Colleges as a mental health innovation. World Psychiatry, 18(2), 141-142. https://doi.org/10.1002/wps.20620

Zabel, E., Donegan, G., Lawrence, K., & French, P. (2016). Exploring the impact of the recovery academy: a qualitative study of Recovery College experiences. The Journal of Mental Health Training, Education and Practice, 11(3), 162–171. https://doi.org/10.1108/jmhtep-12-2015-0052

Zucchelli, F.A., & Skinner, S. (2013). Central and North West London NHS Foundation Trust's (CNWL) Recovery College: the story so far …. *Mental Health and Social Inclusion*, 17(4), 183–189. https://doi.org/10.1108/mhsi-07-2013-0023

Additional File 2: Interview Topic Guides

<u>**Topic guide 1**</u> **– peer tutors – people with dementia and family supporters**

Can you tell me about your experiences of co-producing and facilitating a Recovery College dementia course?

How did you become involved? Why did you become involved?

- *approached by a member of staff*
- *through the central Recovery College*
- *word of mouth or advert*

What have been the benefits of being involved?

What have been the challenges of being involved?

Can you tell me more about your role in co-developing the course?

- *who were you working with?*
- *how often did you meet and where*?

Was there already an idea for the course and how much do you think you helped to develop the course?

Were you happy with the level of involvement you had with developing the course?

Can you expand on that?

What support was provided for you as a coproduction partner?

- *financial*
- *practical (with technology/travel/accessing venues)*
- *emotional*

Who provided this support?

Did you experience any challenges for co-developing the course?

- *practical*
- *emotional*
- *time commitments*

How was your experience of working with the staff to co-produce the course?

- *supportive*
- *different from usual encounters with staff*
- *differences in power relations*

What do you see you gained from being involved with developing the course?

- *e.g. sense of purpose, new skills, confidence in skills, contributing by sharing own experience with others*

From your experience, do you think there are certain skills or motives that have helped you to co-produce the course?

- *confident with sharing experience*

- *willing to contribute*
- *ready to share experiences – can you expand on this?*

Can you tell me about your experience of running the course?

What support do you think you needed to run the course?
- *what worked well*
- *what did not work so well*

What do you think you have gained from running the course?
- *increase confidence*
- *chance to give back*

What do you think people gain from you running the course?
- *ideas that might help people live positively*
- *learning from and sharing experiences with others*
- *seeing someone with dementia leading the course and changing attitudes towards dementia*
- *increased knowledge of resources outside of the course (this could include links with other people)*

Are there negatives for you in running the course?
- *time-consuming*
- *difficult to talk about experiences?*
- *tiring*
- *level of expected involvement may not be in line with actual involvement*

Where and how is the course run?
- *online*
- *face-to-face*
- *what kind of venue?*
- *how often?*
- *for how long?*

Which do you prefer?
- *how do you think this impacts your experience of running the course?*
- *Why*

Which groups of people have you noticed come to the course?
- *people with dementia/supporters/staff?*
- *particular age group?*
- *background/ethnicity?*

Are there particular people/groups that you think aren't attending? Who? Why do you think this is?
- *transport/unable to access online*
- *lack of confidence*
- *aren't aware of the Recovery College*
- *think 'recovery' isn't relevant for people with dementia*
- *do not relate to the people running or attending course so think it is not for them*

How could more people be encouraged to attend dementia courses?

- *changes to how course is advertised (e.g. language/more widely advertised)*
- *buddy system/support with travel/technology*)

Are you involved with evaluating the course?

- *how is the course evaluated? (standard feedback form/questionnaire, more open-ended feedback welcomed?)*
- *is this information used to make changes to future courses?*
- *does it influence whether the course is run again?*

Anything else you would like to add?

<u>Topic guide 2</u> – staff tutors

Can you tell me about your experiences of coproducing and facilitating a Recovery College dementia course?

How did you become involved? Why did you become involved?

What have been the benefits of being involved?

What have been the challenges?

Setting up a course What factors impact the decision to set up a Recovery College dementia course?

- *resources*
- *backing of certain individuals*
- *attitudes towards recovery in relation to dementia*
- *culture within the Trust*

What is the relationship between memory services and the Recovery College?

- *how do they complement one another?*
- *do they have differing aims?*
- *do memory services signpost people to the Recovery College?*

Who is the course open to?

- *people who have accessed services, supporters?*
- *staff?*
- *general public?*

How is the course advertised?

- *social media?*
- *through the Recovery College?*
- *through services?*
- *word of mouth?*
- *any other*

Are there particular groups of people you are aiming to reach with the Recovery College courses and what methods do you use to reach them?

Does this work?

Coproduction How important is coproduction to the course?

- *why is it important?*

What have been your experiences of coproducing the dementia course?

What would co-production of the course ideally look like?

- *is it possible? Why, or why not?*
    - *resources co-producers can access*
    - *time*
    - *decision-making*

- What level of co-production do you consider good enough?

What have been the benefits for you in coproducing the course?

- *ability to work in line with values?*
- *help to re-engage with day-to-day job*
- *professional growth*
- *personally rewarding (how?)*
- *decreased stress/burnout?*

What are the challenges of coproduction?

- *embracing a different power dynamic/valuing each person's involvement? (For you? For the people you're coproducing with?)*
- *amount of time it takes*
- *any difficulties with working with people with dementia specifically? (If yes, what are they, how do you address and overcome these?)*

How do you find people to coproduce courses with?

- *people you've worked with clinically*
- *through the central Recovery College*
- *word of mouth*

Are there certain skills, characteristics, or experience that you look for in a coproduction partner?

- *confident with sharing their experience*
- *willingness to contribute*
- *emotionally 'robust' enough?*

Can you tell me about a time when you were struggling to work in a co-productive way and what you did to address this?

- *the fact that they might benefit from the process*

What support is provided for coproduction partners (people with dementia and supporters)?

- *financial?*
- *practical (with technology/travel/accessing venues)?*
- *psychological/emotional?*

Who provides this support?

Has coproducing the course impacted your day-to-day job?

- If yes, how and how have you managed this? *(Affected interactions with other people with dementia? More likely to apply recovery-focused principles?)*

Running a course Where/how is the course run?

- *online/face-to-face?*
- *what kind of venue?*

How do you think this impacts the delivery of the course?

- *are people with dementia less able to use technology to attend online?*
- *does the course being face-to-face encourage more discussion or building of relationships?*

What have been the positives of running the course?

- For you as a member of staff *(chance to gain holistic view of people with dementia, to see them recovering and thriving, increase personal wellbeing, changed attitudes towards recovery)*
- For peer tutors *(increase confidence, chance to give back)*
- For attendees (*empowerment and self-management, a safe space, learning from and sharing experiences with others, seeing peers leading and succeeding, reduced stigma and more positive sense of self, able to visualise living well, see self as more than diagnosis, ability to build relationships that last outside of the course)*

And the negatives? *(time-consuming alongside other responsibilities)*

Have you felt supported whilst running the course?

- *by the Trust (if applicable) at organisation lead level or manager level?*
- *by fellow coproducers?*
- *by other colleagues?*
- *with administrative staff?*

Who comes along to the courses?

- *people with dementia/supporters/healthcare staff?*
- *particular age group?*
- *background/culture/ethnicity?*

Are there particular people that you think aren't attending?

- Why do you think this is?
  - *transport/unable to access online*
  - *lack of confidence*
  - *aren't aware of the Recovery College*
  - *think 'recovery' isn't relevant for people with dementia*
  - *don't think the course is for them (why might that be?)*

How could more people be encouraged to attend dementia courses?

- *more accessible language*
- *more widely advertised*
- *buddy system/support with travel/technology*
- *ensuring adverts and materials 'speak' to the people trying to reach*

Evaluating a course Do you evaluate the course?

- How? *(feedback form/questionnaire, more open-ended feedback welcomed?)*
- Is this information used to make changes to future courses?
- Does it influence whether the course is run again?

Anything else you would like to add?

<u>Topic guide 3</u> – attendees – people with dementia and family supporters

Can you tell me about your experiences of attending a Recovery College dementia course?

How did you hear about the course?

- *from a member of staff / care team*
- *word of mouth*
- *social media*

Why did you decide to attend?

- *wanted to meet others in a similar situation*
- *wanted to learn strategies to live positively with dementia*
- *wanted to find out what a Recovery College is*

Where and how was the course run?

- *online/face-to-face?*
- *what kind of venue?*

Did you have any worries or concerns before joining the course?

- *getting there / joining online*
- *didn't feel confident enough, worried about talking or meeting others,*
- *what you might be expected to do during the course*
- *how you might feel during or after the course*
- *not sure if recovery is relevant to someone with dementia*

Do you think things could have been done to make the course easier to access?

- If yes, in what way?

How could more people be encouraged to attend Recovery College dementia courses?

- *easier to understand language*
- *more widely advertised*
- *easier ways to sign up*
- *buddy system / support with travel / technology*

Would you prefer courses online or in person?

- Why?

What have been the positive outcomes from attending the course?

- *learning from and sharing experiences with others,*
- *seeing people with dementia running the course*
- *improving how I see myself and my situation*
- *knowing where I might get help or thinking about how I can better manage*

What have been the negative points from attending the course?

How do you think the course could have been improved?

- *ratio between people with dementia / family supporters and staff*
- *did everyone get the chance to speak if they wanted to?*

- *did you see it as a safe space?*

Anything else you would like to add?

<u>Topic guide 4</u> – attendees – staff

Can you tell me about your experiences of attending a Recovery College dementia course?

How did you hear about the course?
- *from another member of staff*
- *word of mouth*
- *social media*
- *through central Recovery College*
- *previous attendance at Recovery College*

Why did you decide to attend?
- *thought learning would be helpful in day-to-day role*
- *wanted to learn more about recovery and dementia*
- *wanted a different way of working with people with dementia*
- *wanted to find out more about the Recovery College*

Were there any barriers to you attending?
- *taking time out of work*
- *permission from line manager*

Or how were you encouraged to attend and why?
- *recommended by colleagues*
- *part of CPD*
- *considering role of coproducer*

Where and how was the course run?
- *online*
- *face-to-face? What kind of venue?*

Did this work for you? Why or why not?

Do you think the course could have been more accessible?
- If yes, in what way?

How could more people be encouraged to attend Recovery College dementia courses?
- *more accessible language (less recovery-focused?)*
- *more widely advertised*
- *support with travel/technology*

What have you gained from attending the course?
- *learning from and sharing experiences with others*
- *seeing people with dementia leading, succeeding, and living positively*
- *reduced stigma and changed attitudes towards people with dementia*

Were your expectations for the course met?

- *what were they?*
- *how and why or why not?*

How do you think the course could have been improved?

- *ratio between lived and professional experience*
- *how the course was facilitated*
    - *did everyone get the chance to speak if they wanted to*
    - *did it feel like a safe space?*

What was your understanding of recovery in the context of dementia before attending? Did this change after attending the course?

- *not recognised as important by colleagues*

Was there any learning that you will take back to your day-to-day role?

Anything you would like to add?

<u>Topic Guide 5</u> – Relating to Equality Diversity and Inclusion

This part of the xx study is about trying to understand what we need to consider in Recovery College dementia courses to best support people from ethnic and cultural minorities with understanding dementia. So this is a very open, exploratory interview – not seeking a particular angle or answer at all, just really interested in hearing your views.

In our prior conversation [*prep for interview*], you mentioned that people from ethnic and cultural minority groups are under-represented in both your memory services and the Recovery College course. So thinking about the whole process and pathway, why do you feel that may be the case? What do you feel is the 'sticking point'?

[You can think about it from the angle of the service users themselves, or other healthcare professionals, or services, systems]

To what extent do you feel that people may feel excluded from services or are choosing not to engage?

Where do you feel that people seek or receive a diagnosis and post-diagnostic support if not from formal health services? Do you know of any other services which pick people up later in their journey?

What do you feel could be some of the unmet needs that ethnic minority people with dementia could have post-diagnosis due to current levels of support, if any?

What do you feel could be some of the wider drivers of under-representation in this group the Recovery College? i.e. geography, socioeconomic, health beliefs of dementia etc

Moving Ahead:

1. What do you feel we in Recovery Colleges and/or the wider health service could do better to attract ethnic and cultural minorities into courses?

2. What do you feel that Recovery Colleges could offer ethnic minority participants that is different or unique to other forms of support?

3. Does your Recovery College currently support or pay for the additional costs of running the courses i.e. travel costs, room, refreshments etc? How is this paid for? Is there anything else that you would like funding for to engage minorities in the course that may be helpful?

4. How do you feel that your current dementia Recovery College courses could or would need to change to better engage and support ethnic and cultural minority people?

Additional File 3: Supporting data on context (C) mechanism (M) and outcome (O) configurations (CMOCs) which support Theme development

Theme 1 Empowering and enabling non-stigmatized identities: When people with dementia who are adjusting their diagnosis attend a Recovery College course that provides them with emotional and peer support (C) enables them to feel more confident and positive living with their diagnosis (M) and they may accept their dementia as part of their identity without stigma (O))

**Context-Mechanism-Outcome-Configuration (CMO)**	**Data example/s**
EVAL CMOC1 If people learn about the types of dementia (C) then they understand it is beyond their control and they are not to blame (M) because they are able to reduce self-stigma as dementia is assimilated and maintained in their identity (O)	CS1-01 attendee with dementia: I felt a lot better when I came out. I went with a guilty conscience and I was frightened to think what I'd done wrong to deserve this, because I don't like the way I am. I thought, "Nobody will like me because of what I've got." I met one or two and I didn't talk to many people because I was scared, but when I came to you, I heard them people talking the way they felt. I thought, "If they've got it, not everybody could be wicked," and I thought, "No, I don't feel wicked no more." I know I've got this disease, but I'm still trying to find out how I got it and what's happening.<br><br>CS2.2-21 attendee with dementia: I mean, it's lovely, I think, for all of us to see her up there and talking to us, and everything.<br>Interviewer: So why is that? Why is that important?<br>CS2.2-21: Well, because I think once you've been diagnosed, you tend to go into your own little world, and some things that I do, I think, "Is that me or is it the dementia?" And, honestly, you don't know sometimes. … I think it's difficult for you people, because there are so many of us that have dementia, but we don't know what type or what the other person is going to … And I can't think of any other way out of it, really. I mean, we've been married 62 years coming up, and it's … I don't know. It seems to have shattered everything, and I don't know how to rectify it because it's me that's causing it.
EVAL CMOC2 If the course provides knowledge about living positively alongside symptoms of dementia (C) it can motivate and empower a sense of agency and control over their futures for attendees with dementia (M) because peer tutors inspire attendees through sharing their personal recovery journey of living alongside their dementia symptoms (O)	Interviewer: So that's a really important thing that you're the same as other people.<br>CS3-03 attendee with dementia: That's right yes. There's a lot of people unfortunately lose their memory. I still cook every day and you know, so I'm not that bad but I am a bit forgetful at times.<br>Interviewer: It's important to keep doing things.<br>CS3-03: And I'm determined to keep cooking every day and I occasionally make a few buns and things. I don't do quite so much as I used to.<br><br>CS1-06 attendee family supporter: it's hard to explain with my mum. It's not that she can't take on board that she's got dementia. I think she's just dismissive of it or doesn't really – you know, but yes, I think now that she's openly said it, I think that we can talk about it and be prepared and try and do all the things we can to help her really.<br><br>CS2.2-30 attendee with dementia: Well, I've learnt a lot from what she was saying about her experiences and the fact that you look around and you see other people that you know that are in the same state. But, yeah, it educated me a bit more about it all, you know what I mean?
EVAL CMOC3 If people with dementia attend dementia courses following their diagnosis and courses are open for people to decide to attend when the time feels right for them (C) then facilitators can create space for	Interviewer: Do you think this is the right time for you two to come along to this type of session?<br>CS2.2-28 attendee with dementia: Yeah.<br>Interviewer: Very soon after you've been given that news.<br>CS2.2-28: Yeah.<br>Interviewer: Why is that?<br>CS2.2-28: Well, just to know there's people out there with it and I am getting the help.

people to tell their story to be validated for the stage they are at (M) because attendees have an opportunity to process their diagnosis with the support of their peers (O)	CS1-02 attendee family supporter: For me, I mean I'm a very logical, very practical person, but sometimes if it all comes at once, you just go, "Hang on a minute, hang on a minute, I've had seven months of nobody being the slightest bit interested." All of a sudden, it all hits you at once. You think, "Hang on, calm down a bit." You need your mind to settle. Sometimes, you need your mind to settle. To settle on the diagnosis, understand what that means and then maybe a month, six weeks afterwards, attend the course when you've got your own head settled down. So I think that's a possibility.
EVAL CMOC4 If the course supports learning about the process of personal recovery together with peers (C) then it increases hope for a more positive message about living with dementia (O) because stigma is broken down in the room (M)	CS1-09 attendee with dementia: You could write on and say how you felt and what you think were causing it and I enjoyed it.<br>Interviewer: What did you enjoy about doing that?<br>CS1-09: Because I could let myself free. I did feel free. I know I'll never get rid of this. I know it.<br>Interviewer: There was a word used in that group wasn't there called 'recovery'.<br>CS1-09: Yes, but you can't –<br>Interviewer: You can't recover from the disease –<br>CS1-09: You can't recover from the disease, but they're right in one way. How can I put it? You can recover in yourself. The things you've thought are not true. So you know in your mind it's not you.
EVAL CMOC5 If attendees can learn the importance of maintaining connections with important people and activities in their lives (C) then this can help maintain their sense of identity, meaning and purpose (O) because the course generates opportunities for talk about self to reinstate identity (M)	CS3 observation fieldnotes: talk about animals about what makes them stressed.<br>CS2 the FS thinking about birds and doing this activity with husband.<br>Interviewer: Because she talked about some of the difficulties, didn't she, and she talked a bit about the sort of strategies that she'd come up with to cope. And I liked it because I think you laughed when she said *"taking the doors off".* She got the screwdriver out, didn't she, and took the cupboard doors off in the kitchen? [so she could find things more easily]<br>CS2.2-30 attendee with dementia: Well, I was in the building trade, you see, and I was a bricklayer/ plasterer.<br>Interviewer: Oh, that's why it made you laugh.<br>CS2.2-30: But I always wanted to be a joiner. And when I was when I was a young lad, this neighbour, her husband was a plasterer and he said, *"Oh, I'll take you for this interview [unintelligible]*." He said, *"I know [name]. He runs this apprentice scheme."* I sat down at the table and he was at the other side, *"Now then, young man," [unintelligible], "Now then, young man,"* he said, *"What do you want to be?" "Oh," I said, "I want to be a joiner."* And I did, I wanted to be a joiner because I was always good at woodwork.

Theme 2 Managing uncertainty for positive reframing: When tutors are skilled in working together, balancing input, and responding sensitively to verbal and non-verbal cues, especially when attendees express a range of emotions such as distress, tearfulness, or self-blame when recalling their diagnosis or on seeing others' distress (C) attendees feel emotionally supported and included (M) •meaning people feel able to remain in the Recovery College Session (O)

**Context-Mechanism-Outcome-Configuration (CMO)**	**Data example/s**
EVAL CMOC6 If people with dementia express a range of emotions when recalling their diagnosis or seeing others' distress (C) then skilled tutors who support each other can identify and diffuse situations in the moment and validate that person's experience (M) this creates a safe environment where people are able to share their experiences (O)	CS1-02 peer tutor with dementia: It just makes it easier to understand and to find a solution to it. So I think in that respect, I'm quite a useful person to that team as well because if I say so myself, I've got good interpersonal skills. I can diffuse a situation. I can give someone a bit of love if they need it. I can be a little bit stern if they need it and I can make them smile if they need it, and that's important to lighten the load on them.<br><br>CS1-02 attendee family supporter: So I was worried that it would all get a bit over-emotional and I'd end up going, "Do you know what, I've got to get out of here," but it didn't at all. But when it did get emotional for a couple of people, it wasn't the sort of emotion that we couldn't handle. Yes, so this lady being upset, it was handled beautifully by the staff there and the doctors there. …you will actually find, because I think I found there, that everyone there supported people that were upset. It wasn't they were just supported by the presenters, but actually, other people in the room supported them as well.
EVAL CMOC7 If staff and peer tutors can balance input from course attendees by redirecting back to others (C) then individuals won't dominate the conversations (M) enabling everyone to speak, contribute and not be interrupted (O)	CS3 observation fieldnotes: [the session] moves to a post-it note activity. We move to sit in groups of three which means there is a staff tutor, peer tutor or [researcher] with each dyad. Staff Tutor-02 gives out post-it notes and pens, the flip chart says 'What causes you stress?' We are asked to write down things that cause us stress. CS3-02 attendee family supporter says her husband struggles to decide on things, so when they go out for a meal, they look at the menu online beforehand so he can choose and not be stressed at the event. CS3-01 attendee with dementia [her husband] interjects that he does this, but mainly to see if the food looks good and if he wants to go, then complains about food when eating out. Staff Tutor-01 redirects back to CS3-02 and says what a great idea, think of other things for next week.<br><br>CS3-06 peer tutor family supporter: One person on one previous course was very much, "I'm going to dominate the course and I'm going to tell you everything that's happened to me." And actually that's not helpful because everybody needs their own chance to speak and their own time to share their experiences and if somebody was just railroading in every time with me, me, me, that probably wouldn't help. I think the general thing is being aware of other people wanting to help them and empathetic to what they're doing. You need to have that empathy with what they're going through.
EVAL CMOC8 If there is too little time to cover planned content and the session falls behind (C) tutors may feel rushed to move things on (M) and this limits interactive time shutting down time for the attendees to contribute or to respond to emotions (O)	CS1 observation fieldnotes: The group are told this is time to think about concerns and Slide 12 indicates this is a paper exercise. The staff tutor moves to a chair next to an attendee with dementia as the family supporter peer tutor stands to lead this section, and asks if people are willing to share their score and then they will talk about concerns. CS1-09 attendee with dementia says *"I am confused"* and a family supporter attendee says in response *"oh bless you"* then says she feels frustrated and angry with her parents for not making arrangements, so now she cannot access parents business accounts and is waiting for a court of protection order. Another family supporter attendee asks her what to do if you can't get power of attorney and she explains why not in her circumstance. The staff tutor says that the family supporter peer tutor will cover that topic later. It appears she is trying to move the activity on. I note several times during the activity she says *"We need to move on, we are running a little behind time".* My [observer] impression: an urgency to stick to time meant that when attendees were talking and sharing experiences these

	conversation tended to be halted.<br><br>CS4 observation fieldnotes: With minutes left, CS4-02 peer tutor family supporter introduces the issue of driving and dementia, telling DVLA and claiming Attendance Allowance, then *"I think that's about it".* There is a sense of having to get all the information covered, even if done in a bit of a rush.

Theme 3 Connecting together enabling hope: When the person with dementia is empowered, often by family supporters, to engage in personal recovery activities such as peer tutoring or attending a course (C) they are able to connect with, and learn from, others with similar experiences (M) meaning people with dementia have a safe place to discover new meaning and value in their relationship and experiences (O)

**Context-Mechanism-Outcome-Configuration (CMOC)**	**Data example/s**
EVAL CMOC9 If people with dementia attending are offered extra help and time for greeting and settling in (C) then social nervousness can be abated to some extent (M) because attendees are more relaxed and connect through talking more amongst themselves (O)	CS3-03 peer tutor family supporter: The reason I say that is that the person with dementia is probably very anxious and very nervous. And if he or she has somebody with them who they know and love, that can only help them to settle down. I think that's the right thing to do. Sometimes it's difficult. We had one lady who came on her own, all you've got to do is make a little extra effort with her to make her feel comfortable because she hasn't got anybody alongside her. It is easy to do, all you have to do is chat, it's all about talking.<br><br>CS2.2-13 attendee with dementia: I found it interesting. I enjoyed it. Yeah. It was enjoyable…It's just something different, you know… Just something different, yeah. Very friendly. Yeah.<br><br>CS3-03 attendee with dementia: I think you feel apprehensive going and self-conscious about going don't you but I think it does you good. … I didn't worry about going. I might have been apprehensive a bit the first week. You are aren't you, but – anything to help isn't it. And make you realise you're not alone. There's loads of people like you. It's not the end of the world.<br><br>CS1-09 attendee with dementia: Yes. I was a bit concerned wondering, *"How can they help me?"* and, *"Will I be all right or will it change my life?"* but yes, it has. It's made me feel not guilty no more.<br>Interviewer: So you talked about that change in how you felt, being about meeting other people with dementia in the room who were talking about their experiences. Was there anything else about the information that was being discussed in the room?<br>CS1-09: Well, I felt warmth.<br>Interviewer: So you felt warmth, a sense of warmth.<br>CS1-09: Warmth and wanted, like they understood me and I thought, *"Well, somebody understands what I'm going through,"* and that means a lot to somebody like me.
EVAL CMOC10 If the course begins with a warm-up activity that is non-dementia related (C) attendees do not feel compelled to share anything emotive or identify their dementia status (M) because the course is seen to be a positive and inclusive shared learning environment (O)	CS3 observation fieldnotes: Staff Tutor-01 had made a series of slides with animals and questions all related to the animals people had spoken about in the warm-up activity from the session last week. The first slide asked *"Who has a goldfish?"* No-one answered until ST-01 looked at the dyad CA-03 person with dementia and CA-04 family supporter saying *"I thought you said you had a goldfish?"*. CA-04 said no it was their children who had had a goldfish, ST-01 responded *"Ah you had a goldfish in the house".* CA-03 then spontaneously spoke and said they couldn't have a cat because of the road. It was noticeable that in this session, CA-03 spoke much more within the group with snippets of her life.<br><br>CS3 observation fieldnotes observer impressions: Set up that warmed people up for sharing and did not put people in a position that they could not contribute to, or in a category.<br><br>CS3-01 attendee with dementia/dyad with CS3-02 family supporter interviewed together. Family supporter includes spouse in her comments. Interviewer: And one of the things I noticed was that at the beginning of every session we talked about animals or your crows and things like that.<br>CS3-01: Oh right, I know the one you're talking about. That was neat that was. You could say something and have a little laugh you know.

	CS3-02: We've always been very open haven't we?<br>CS3-01: Yeah, in a way we've either got it or we haven't basically. But not saying that I don't want to bother about it, I'd like it all to come back again.<br>CS3-01: – to get there. Now I made them laugh when they were on about the stick and things. I said, *"Well I'm sorry but I didn't know about that. I can tell you about these pigeon things."* To get out of the way of what I'm not getting. [Laughs]
EVAL CMOC11 If there is time for informal conversations, for example during ice-breakers and tea breaks (C) course attendees can better connect with each other (M) because informality contributes to creating a safe space for talking more openly during course sessions (O)	CS3 observation fieldnotes: During coffee break CA-03 and CA-08 attendee family supporters were chatting together about CA-03 having high standards but can no longer do housework to the standard they want. CA-08 gave a sense of understanding and empathy. I think they probably bonded after recognizing a shared interest in group conversations.<br><br>CS1-02 peer tutor with dementia: We could have done that through an ice-breaker and that would have got people talking a little bit about themselves. I always have an ice-breaker if I do a presentation. I think that works wonders for people. It relaxes them a bit and you can find out if there is anything that's really bugging them. Also, who the loud mouths are, etc.<br><br>CS3-01 attendee with dementia: Oh right, I know the one you're talking about. That was neat that was. You could say something and have a little laugh you know.<br><br>CS3-02 family supporter attendee: I felt very comfortable, I think because we had tea and coffee when we went in and what not.<br><br>CS1 observation fieldnotes: I ask *"This is quite a large group?"* and she says yes and they are more talkative than usual groups explaining usual groups sit and listen then talk during the discussion bits. There is generally chatting during the coffee break.<br><br>CS3-06 peer tutor family supporter: I just think in the room everybody can easily see people that haven't had a chance to speak yet. CS1-01 peer tutor with dementia will draw somebody in and say, "How do you feel about that?" I think you'd miss that on Zoom. Also it may be a bit of a bizarre thing but you'd miss the coffee breaks and we have really good conversations in coffee breaks. We will find out, we'll talk to people and people are open and much more likely to say something that's concerning them or something they might think it's silly and don't want to say in front of anyone else. All sorts of things have come out of previous groups in the coffee break haven't they. We'll talk about all sorts with people and you'd miss that.<br><br>Interviewer: Yes, that's one of the things I noticed [while observing] at the session, that there was a lot of chat at the beginning when we all got a cup of tea before we sat down. And then a nice long break in the middle for a chat and everybody was moving around and talking. And that felt comfortable, people felt comfortable.<br>CS3-03 peer tutor family supporter: It gave people time to think as well.
EVAL CMOC12 If there is space for discussion and debate in the wider session and/or in small groups (C) attendees can learn things from each other (M) this brings about higher levels of interaction and shared engagement with the learning materials (O)	CS2.2-12 attendee family supporter: It's just that we didn't have much debate. I mean, there was a comment made, wasn't there, that sometimes there's a lot fewer people in the room and they have discussion groups. And going back in my experience, the things I've been involved in historically, that was always useful to have discussion groups going on around tables and, you know, feedback coming from the discussion groups on issues. And I think that you probably use that, don't you?<br><br>CS3 observation fieldnotes: [learning material – 10 minute video] Video made by [name of peer tutor with dementia not present]. Introduced by lead staff tutor and acknowledged that this person and their wife had developed the course but were no longer able to attend as too busy. Gave overview of the video and instructions for what to look out for, preparing people for the group discussion and enabling them to contribute. Video discussed the reality of diagnosis with some very emotive material shared and dark places that [name] had been to. Then the video became more positive as [name] reported finding his purpose. Co-lead staff tutor then led

	discussions by inviting reactions to the video that were recorded on a flipchart. 11 (her) smiles and shares thoughts about findings purpose which lead staff tutor builds on. 2 is very emotional after the video despite having seen several times in previous courses. She discusses her reaction with the group which the staff tutors help to defuse. They acknowledge the difficulties. 10 is checking the flip chart. 7 emphasizes the need for self-motivation and thinking differently, explaining what the challenges are for her and how she needs to think differently about how to go about things she enjoys, maybe participating in a different way. This encourages an exchange between some of the members of the group who are validating each other's experiences. There is quite an exchange where the group acknowledges that the person doesn't change but need to change what/ how involved in things including 13 who didn't always overtly participate.<br><br>CS4-01 staff tutor: We shortened it to make it more discussion based. Because it was online we had to miss out some of the stuff that we'd have done in a face-to-face group. But I think that was good. And now, when we bring it back face-to-face, [we] probably won't add a great deal to create space for discussions. … The Recovery College model, you know, the actual ethos of them, we actually, you know, we get, you know, the experts by experience and the people in the Recovery College saying "This is what they need," so we shortened it and that worked out really well. So it did change in that way but that was a positive thing. Because I think sometimes as professionals we hide behind presentations and PowerPoints. I think as I've become older and more experienced, I put less slides in so they've time for the discussion and I run the slides off. I think that was quite beneficial really.<br><br>CS1-02 peer tutor with dementia: A lot of people there had stories that they wanted to tell, they were bursting to tell and we didn't have time to listen to them. Listening to them is as important as telling them, and I know we weren't there for a therapy session, but look, anything like that tends to turn into one. Everybody there has got a story and they want to tell it, and if you don't let them, the danger is that they'll lose interest in what you're trying to say, because really, there might be umpteen different types of dementia out there, but it's a lot to take in, especially if you've only just been diagnosed a week or two before.
EVAL CMOC13 If people with dementia find it difficult to actively contribute to the course through conversation or activity (C) then they may still experience a sense of connectedness with others and the learning activities (O) because they are watching and listening (M)	CS1 observation fieldnotes: CS1-07 attendee with dementia sits quietly looking forwards and sipping cup of tea. … is looking ahead there is no emotion on her face but she seems to be listening. … I note that CS1-07 is awake at all times and looking ahead but is not contributing at all.<br><br>CS3-01 attendee with dementia/dyad with CS3-02 family supporter interviewed together. Family supporter includes spouse in her comments. CS3-02: But I watched CS3-01 to make sure he's OK and the thing I did notice which I felt was really important, he had his attention the whole time. You were really engaged with it. So it must have been of interest to CS3-01 because there wasn't a time when I saw him zoning out, which you can do sometimes. So you were very comfortable there weren't you.<br>CS3-01: Yeah it was fine being there all the time. If halfway through it you think, "Oh god we've got to get on with this again, blah, blah, blah," then they're likely to squeeze out through the door or something or start saying –<br>CS3-02: That didn't happen did it.<br><br>Interviewer: You said that you really enjoyed meeting other people in the same situation as you. Did it change the way in which perhaps you thought about your own diagnosis at all? Did it change the way in which you thought about things?<br>CS3-03 attendee with dementia: No I don't think so, that was just nice. I suppose it's just nice. There was a group of people there that are the same as you.<br>Interviewer: Was there anything you didn't like at all about the course where we were all sitting round the table and the videos were playing and people were talking and that type of thing? Anything you didn't like?

	CS3-03: No. I'm pretty easy going really. I just take it as it come really.<br>Interviewer: That's great.<br>CS3-03: It's all done for your benefit, that's what I think, to help you. Perhaps some things might not apply to you so much as others. But you have to hear the whole thing don't you.<br><br>CS2.2-13 attendee with dementia: I found it interesting. I enjoyed it. Yeah. It was enjoyable.<br>Interviewer: What about it was enjoyable?<br>CS2.2-13: It's just something different, you know. Just something different, yeah.
EVAL CMOC14 When people with dementia attend courses co-facilitated by a peer tutor who also has dementia and who focuses on reclaiming a positive life after diagnosis (C) this helps people believe there is hope when living with dementia (M) because connecting with peers who share lived experience fosters hope and optimism about managing dementia positively (O)	CS3-03 attendee with dementia/dyad with CS3-04 family supporter interviewed together. The person with dementia includes spouse in her comments.<br>CS3-03: The main thing I thought was it would be nice to meet other people who are in the same position as me, whose memory is not as good you know. You know you've not got two heads or anything like that sort of thing. I mean that's why I thought that was nice. My main reason I thought that was nice, that's good for CS3-04 as well to meet others, for both of us really. See how other people cope with it. I mean I'm glad I went, that was – I needed to meet other people with the same.<br>Interviewer: So did it meet that, there were a few other people living with memory problems, was that helpful for you?<br>CS3-03: Yeah, I suppose the general atmosphere was the same really wasn't it. You felt like a group of people in the same position didn't you.<br>Interviewer: Yes, that word atmosphere is quite interesting. How did it feel for you then?<br>CS3-03: Yeah, you feel a bit apprehensive when you go the first time. Otherwise I didn't really worry and I didn't notice any tension, did you CS3-04?<br>CS3-04: No
EVAL CMOC15 If people with dementia attending a course can see and hear other's lived experience of dementia (C) they learn they are not alone (M) therefore people experience a sense of connection, belonging and solidarity (O)	CS1-09 attendee with dementia: As I said, when I came there, I was scared. I really was scared and I didn't know what to do. I was listening. It was very interesting and then I thought, "Right, I will have my say and I will tell them." Now I met people there, I know all them, what's got dementia –. … Because I wanted help. I wanted to put my mind clear, what I thought dementia was, and I want to put that clear, and then when I met these people there, I knew it wasn't my fault. I haven't done nothing wrong and I've got that to thank for, and I told my son.<br><br>Interviewer: So was there anything from this morning that's helped you particularly. Was there anything that stood out?<br>CS2.2-30 attendee with dementia: Well, yeah, listening to her mainly talking about her experiences, and that, and, well, the fact that I looked out and I could see there's others that are obviously in the same boat as me, as you might say.<br><br>CS3-03 attendee with dementia: The main part that I like is it was really seeing how people like me have got bad memory's. I think that does me more good, I don't feel so, you know. It's mixing with people isn't it. You don't know them do you but you sit near them and you chat don't you. I think that is a good tonic. … To go and you know have other people, you're not such a freak you're just a bit of memory loss. Which when you're nearly 90 what do you expect?
EVAL CMOC16 If the peer tutor with dementia connects with attendees who may be shy, embarrassed, or face cognitive challenges (C) then attendees will be more comfortable participating and sharing their experiences (O) because attendees are empowered to contribute (M)	CS2-02 peer tutor with dementia: Well just it gives me a chance to talk. It gives me a chance to show people that people with dementia can talk. Again when I'm a room I can ask people, "What did you expect when you came in?", it's not quite the same online. You know, "What were you expecting me to be like knowing that it was somebody with dementia delivering the course?", it doesn't work the same. I don't get the same feedback as I do face to face. But I wouldn't do something if I didn't love it.<br><br>CS1 observation fieldnotes: A powerful moment was when the staff tutor called the group together to enable the voice of the person with dementia who was previously distressed to be heard, describing how different and better she felt having joined

	this group today. The person makes the statement *"before I came here I was blaming myself - now I am not blaming myself"*.<br><br>CS2.2-22 attendee family supporter: The honesty of what people said. There was only a couple of people mentioned things – a gentleman at the front about his father. The honesty of how it came out, how it came over, it's good, it gets something off your chest. And I probably should have said something myself, but it's difficult. How do you handle it? And I don't know to ask the question, to be truthful. I didn't want to let myself look a fool, and I'm not really clever enough. I've never been clever enough in my life to … I've achieved some things, but not to the heights of what my wife has. I think we've all done it in our own way, haven't we? I've travelled part of the world and got on with my job, but to stand up in front of a group of people and asked a question that they might think silly, it frightens me, so I didn't bother.
EVAL CMOC17 If people with dementia are struggling and still adjusting to their diagnosis (C) peer tutors genuineness through sharing their own lived experience can quickly connect with attendees (M) this fosters hope for a positive life with dementia (O)	CS1-02 peer tutor with dementia: I believe that my dementia, my diagnosis of dementia and the life I'm living now has given me a perspective that is useful to Recovery College. I think it's gifted me with, my lived experience has gifted me with the ability to actually speak to people who have just been diagnosed with a voice that is unique and can't be – I can tell them things that a practitioner can't tell them and I can get away with it because I'm there, I've been there. I'm in the same boat as them. And I think that, so I can be a role model even, I dread to think I've ever been one of them, but I think I can be because here I am standing up in front of them.<br><br>CS2.2 observation fieldnotes: The session then began with the peer tutor with dementia introducing herself and talking at some length through her lived experience of being diagnosed with young-onset dementia and her view that clinicians know so little about living with the condition "I was so shocked at the lack of awareness" "I was so shocked about the negative manner of the diagnosis process – all about negative language". The neurologist said "there's nothing we can do". "Do not underestimate the words and body language used". She talked about being given a sense of "no hope" but wants to change the narrative to focus on what people can still do, not what can't. talks about the lack of, at the point of her diagnosis, the word hope, in any of her conversations or written information. She thinks the term 'living well' is a bit of a problem, as it sets a high standard, might not be achievable for many, on every day, or at all, as living with dementia brings despondent days; "it's a bummer, but we can still enjoy life if we concentrate on the better/good days. Again, we need that little 4-letter word – HOPE – we need to hear this". (Researcher reflection: I also wondered if this was a message about acceptance and being kind to yourself on the 'bad' days). This is described as being in relation to being offered a more hopeful message about living with dementia at the point of diagnosis. The attendees are listening and focused.<br><br>CS2.2-30 attendee with dementia: Well, mainly listening to what she was saying and what her experience is, and that…Well, it makes me think of if I get worse, which I maybe will do – we don't know – it gives me a general idea of what to expect. You know what I mean?<br><br>CS2.2-31 attendee family supporter: I tend to go into these things with a completely open mind, so I didn't know what I was going to see and certainly Dad wouldn't have known what he was going to see, but I just thought that there would be, some positives would come out of it, we would hear about things that we didn't know about and maybe be able to contribute with things that might help others.<br><br>CS3-05 peer tutor with dementia: I don't think of myself as a tutor, no, I'm just somebody who hopes to help people. I just want to – when we first got the thing. [CS3-01] staff tutor went in and she was chatting away and I thought, "Well what's next." And then that did come out didn't it, that there is a next, but that was a little bit longer because they'd got to put the whole thing together hadn't they. So I think

	that's lovely.
EVAL CMOC18 If peer tutors with dementia share their own experiences of finding ways of living positively with dementia despite the difficulties (C) this resonates with and empowers attendees (M) and there is connection and shared learning (O)	CS1-02 peer tutor with dementia: I want to fire people up with enthusiasm and show them the benefits of doing all of these things and how they will impact their future and also how it will help their loved ones and their circle of friends and families later on. These are the decisions people put off and don't want to face, but they must face. So that's the important thing for me. … I also see a role for myself as someone with lived experience, someone who's living with dementia but is still able to speak. Speaking to professionals too to say, you know, "This is me. I'm not someone that you do things to. I'm not an object to pity. I am a reasoning, functioning human being. Sometimes I have trouble expressing myself, but here I am," just to let people know that sometimes the person that they perceive someone to be is not that person at all. We are compos mentis.<br><br>Interviewer: What did you think about his presence as a trainer and a tutor there on that group?<br>CS1-09 attendee with dementia: I thought it was good because it's going to show … I've got to put this right. It's going to show the dementia people that they can do things. I mean, a lot of people think, "Well, I can't do nowt' because I've got dementia," like me, but when he spoke and he said he was doing it, and it was like I thought, "You must have been really brave," and that's showing us lot that we can do things ourselves as well. We don't need to depend on everybody.<br><br>CS1-09 attendee with dementia: You don't need to think, "Oh, I've got to have you to help me because I've got dementia."<br>Interviewer: So seeing him in that position, standing at the front of the room and –<br>CS1-09: And talking and that.<br>Interviewer: Talking and presenting, yes.<br>CS1-09: Yes, I thought he was very brave. I don't know if it's helped anybody else, but it's made me think, *"You can do things yourself if you want to, but you've got to put your mind …"* It's like knitting. Some people wouldn't knit because they don't put their mind to it. You put your mind to something and try to block this out, it can help and I've never been so happy since I've been there.<br><br>Interviewer: Anything else that you've heard this morning that sort of rung a particular chord for you?<br>CS2.2-28 attendee with dementia: Just about everything, really. I just sat and listened and ... No, it was very encouraging, I thought, but I'm not going to skydive. [Laughs]<br><br>CS2.2-29 attendee family supporter: I found that [peer tutor with dementia] concentrating more on the positive side and how to do things … I wasn't quite sure at first whether it would be all doom and gloom and, really, I thought if it's going to be like that, I didn't want my mum to come, but I wanted to know about it. But I found it so positive. It was so nice and I'm so glad that my mother did come because it's sometimes second-hand information if I'd have told her, she probably wouldn't have believed me. Yeah.<br><br>CS2.2-07 attendee staff: There was a man who asked a question about how dementia can affect your speech, and I know this is something that has impacted [peer tutor with dementia] when she was giving the talk. They had a conversation, and it was really nice to see the connection that they could make with each other and how they could understand where the other one was coming from because it's something that impacted them both, obviously in different ways. I don't know, it was just nice to see that this man felt seen and heard by somebody who is actually experiencing what he's experiencing.

EVAL CMOC19 If an attendee does not recognise their experiences in the course content and discussions (C) then attendees may not feel they have learned anything (O) because they find these difficult to relate to (M)	CS4-01 attendee family supporter: It was good talking to other people but I think some people’s parents or partners were more advanced than mum. And I’m not sure, it was interesting, but I’m not sure I got out of it really anything for me, yeah.<br><br>CS2.2-21 attendee with dementia: And I just had nothing but admiration for her because you can see by the way she’s going that it’s beginning to affect her a lot more, but she knows exactly what she wants to get over and how. I think she’s a wonderful lady, but I cannot take much away because I don’t know what sort of dementia she has, but it doesn’t seem as if it’s my sort. … I obviously didn’t bring it out yesterday about the hallucinations that I get. I don’t think I recall her at any time saying that she had that sort of thing. … But I would love to meet somebody that does [laughs] and how they get around it or what they do. Because sometimes on a night, it’s quite frightening. … I don’t know what I expected out of yesterday. And I suppose seeing [peer tutor] there, sort of, “Oh, [peer tutor].” [Laughs] So it sort of calmed me down a bit, but I still come away thinking, “What have I got?” … I don’t know. I found that I didn’t think it related to me specifically or my type of dementia specifically, but I look around and I think, “Where do I fit in?” And it is difficult to know where you fit in in those cases. I would have liked it to have come out and smacked me around a bit and said, “This is where you fit in,” but it didn’t happen. … Well, I don’t know how, because if you’re … I mean, we’re all different, aren’t we? So even if you had us all sat together and said, “Oh, that group there, they’ve got dementia,” it still wouldn’t work.
EVAL CMOC20 If course materials include culturally diverse stories, references and activities (C) then this will support discussions, make meaning of and increase acceptance of dementia in cultural minority groups.	CS1 staff tutor: I do have used particularly, you know, coming from ethnic minority myself. I would. I would like, you know, people from my background or other ethnic groups to participate. … And I'm not certain whether we are actually including within the teams sort of cultural competency. I think I do it on an individual basis.<br>CS4 CA-01 attendee family supporter: My mum remembers when she's younger. I can't share [these memories] with her because I mean, I know you can't share with what you've only heard. But if my mum was born in [Caribbean], we were in [UK city] and I was born there and now we live [UK town]. I could probably take her to [former UK city] but, I can't take my mom to [Caribbean] and take her around. I raised that in the course that aspect of not being able to share those memories [as they advised].
